# Pathogenesis of Rift Valley Fever Virus in a BALB/c Mouse Model Is Affected by Virus Culture Conditions and Sex of the Animals

**DOI:** 10.3390/v15122369

**Published:** 2023-11-30

**Authors:** Victoria A. Graham, Linda Easterbrook, Emma Kennedy, Emma Rayner, Stephen Findlay-Wilson, Lucy Flett, Emma Louise Wise, Samantha Treagus, Susan Fotheringham, Sarah Kempster, Neil Almond, Stuart Dowall

**Affiliations:** 1 UK Health Security Agency (UKHSA), Porton Down, Salisbury SP4 0JG, UK; victoria.graham@ukhsa.gov.uk (V.A.G.); linda.easterbrook@ukhsa.gov.uk (L.E.); emma.kennedy@ukhsa.gov.uk (E.K.); emma.rayner@ukhsa.gov.uk (E.R.); stephen.findlay-wilson@ukhsa.gov.uk (S.F.-W.); lucy.flett@ukhsa.gov.uk (L.F.); emma.wise2@ukhsa.gov.uk (E.L.W.); samantha.treagus2@ukhsa.gov.uk (S.T.); susan.fotheringham@ukhsa.gov.uk (S.F.); 2 Medicines and Healthcare Products Regulatory Agency (MHRA), Blanche Ln, South Mimms, Potters Bar EN6 3QG, UK; sarah.kempster@mhra.gov.uk (S.K.); neil.almond@mhra.gov.uk (N.A.)

**Keywords:** arbovirus, Rift Valley fever, mosquito-borne, animal model, preclinical, development, pathology

## Abstract

Rift Valley fever virus (RVFV) is a mosquito-borne zoonotic pathogen causing disease in livestock and humans. Whilst initially restricted to the African continent, recent spread to the Arabian Peninsula has highlighted the likelihood of entry into new regions. Due to the absence of a regulatory-approved human vaccine, work is ongoing to develop and assess countermeasures. As such, small animal models play a pivotal role in providing information on disease pathogenesis and elucidating which intervention strategies confer protection. To develop and establish the BALB/c mouse model, we challenged mice with RVFV grown from two separate cell lines: one derived from mosquitoes (C6/36) and the other mammalian derived (Vero E6). Following infection, we assessed the clinical course of disease progression at days 1 and 3 post-challenge and evaluated viral tropism and immune analytes. The results demonstrated that RVFV infection was affected by the cell line used to propagate the challenge virus, with those grown in insect cells resulting in a more rapid disease progression. The lowest dose that caused uniform severe disease remained the same across both virus preparations. In addition, to demonstrate reproducibility, the lowest dose was used for a subsequent infection study using male and female animals. The results further demonstrated that male mice succumbed to infection more rapidly than their female counterparts. Our results establish an RVFV mouse model and key parameters that affect the course of disease progression in BALB/c mice.

## 1. Introduction

Rift Valley fever virus (RVFV) is a prototypic phlebovirus species, classified in the *Phenuiviridae* family within the *Bunyavirales* order [1] and is the causative agent of the zoonotic disease Rift Valley fever (RVF). RVF was first described in the Rift Valley of Kenya in 1931 when a fatal infectious disease broke out among sheep [2]. The virus is transmitted via mosquitoes, including those from the *Aedes* and *Culex* genus, and thus has significant potential to expand its geographical range. Since the 1950s, regular pandemics have occurred throughout Africa [3], but since 2000, it has expanded into the Arabian Peninsula with outbreaks in Yemen [4] and Saudi Arabia [5]. In livestock, especially sheep, it generates abortion storms in pregnant animals [6] and results in a high mortality rate in newborn lambs [7]. In humans, the disease can cause mild flu-like symptoms, hepatitis, retinitis, lethal encephalitis and haemorrhagic fever, with the overall mortality rate being 0.5 to 1.0% [8].

Due to the severe disease and high potential to spread into new regions, RVFV is included in the WHO R&D blueprint list as a priority pathogen [9]. Presently, there are a small number of veterinary vaccines available in a limited number of African countries, but for humans, there is no fully licensed commercial vaccine to date. Work is ongoing applying a number of vaccine strategies [10].

To establish the preclinical protective efficacy of candidate vaccines, testing is required in appropriate model systems. For pathogens where disease outbreaks are not amenable to undertake Phase III, regulatory approval for vaccines and therapies may be sought by evaluation in at least two species via the Animal Rule regulatory pathway [11]. Small rodent models are often the first animal model assessed. Murine infection studies for RVFV were first described in the 1950s, when the susceptibility of wildtype mice was reported following challenge using intracerebral and intravenous routes [12]. They have continued to play an important role for studying RVFV pathogenesis and assessing protection afforded by interventions.

As with any modelling system, there are a variety of factors which can intrinsically and extrinsically affect outputs. For example, during the development of vaccine and immunotherapies, it is of increasing importance to consider sex as a biological variable within studies [13]. The mechanisms underpinning sex differences to viral infections are not fully ascertained and could involve a plethora of roles including immunological, hormonal, behavioural, epigenetic and genetic factors. The source of challenge pathogen may also exert an effect, possibly by selection of genetic variants or post-translational modification of virion proteins. With zoonotic arboviruses, such as RVFV, that infect both arthropod and mammalian hosts, they can be cultured in a variety of host cells from different species. These may thus have an effect on subsequent infection kinetics; indeed, it has been shown that short glycans, such as paucimannoses found on the surface of insect cells, have an important role in the host immune response [14]. By contrast, mammalian cells often provide more complex and hybrid glycans in addition to those such as oligomannose glycans arising from insect cell systems [15].

To establish a mouse model of RVFV at the UK Health Security Agency (UKHSA), we grew RVFV in mammalian and insect cells to elucidate differences in infection outcomes. For these studies, we used the strain ZH501, isolated in Egypt in 1977 from a patient with fatal haemorrhagic fever [16]. This strain has been used widely in RVFV studies, including in over 30 studies of vector competence (reviewed in [17]). In addition, we also compared the outcome of infection in female and male mice to establish whether sex differences were observed.

## 2. Materials and Methods

### 2.1. Ethical Statement

All experimental protocols with animals were undertaken according to the United Kingdom Animals (Scientific Procedures) Act 1986, with studies conducted under the authority of a UK Home Office approved project licence. The experimental protocols were approved by ethical review at Public Health England (PHE) by the Animal Welfare and Ethical Review Body (AWERB) on 15 July 2021 (Approval Code: PPL P82D9CB4B). This research is reported in accordance with the ARRIVE guidelines (https://arriveguidelines.org, accessed on 1 March 2022). Prior to the start of the study, humane clinical endpoints were set which consisted of 20% weight loss, compared with baseline; inactivity/immobility; neurological signs; or on the advice of severe disease from the Named Animal Care and Welfare Officer (NACWO).

### 2.2. Animals

BALB/c mice, aged 6–10 weeks on arrival, were obtained from a UK Home Office accredited facility (Envigo RMS UK Ltd., Oxford, UK). Animals were randomly assigned to groups and were housed in cages in groups of 5 designed in accordance with the requirements of the UK Home Office Code of Practice for the Housing and Care of Animals Used on Scientific Procedures (1986). During and after challenge with RVFV, all procedure, housing and husbandry took place inside a flexible-film isolator housed within a Containment Level 3 facility. Food and water were available *ad libitum* and environment enrichments were provided within the cages.

### 2.3. Cells

A mammalian cell line derived from an African green monkey, Vero E6 (Product 85020206; European Collection of Cell Cultures (ECACC), Salisbury, UK) was cultivated using Dulbecco’s Modified Eagle Medium containing GlutaMAX (DMEM; Gibco, Paisley, UK) supplemented with 10% foetal bovine serum (FBS; Gibco, Paisley, UK). Cultures were grown at 37 °C in a 5% CO_2_ humidified incubator.

A mosquito cell line derived from *Aedes albopictus*, C6/36 (Product 89051705; ECACC) was cultivated with Eagle’s Minimum Essential Medium containing GlutaMAX (EMEM; Gibco, Pasiley, UK) supplemented with 10% FBS (Gibco, Paisley, UK) and 1% non-essential amino acids (NEAA; Sigma, Gillingham, UK). Cultures were grown at 28 °C.

### 2.4. Virus

RVFV strain ZH501 stock (mouse brain suspension; passage 10) was propagated on Vero E6 cells at 37 °C for 3 days to produce an initial stock. To compare growth in mammalian and insect cell lines, the virus was passaged three times in Vero E6 and C6/36 cells, respectively.

### 2.5. Sequencing

Tissue culture supernatant from RVFV passaged three times in Vero E6 or C6/36 cells was sequenced, in duplicate, to determine consensus genome sequences and identify differences between the mammalian- and insect-grown viral genomes. RNA was extracted using a QIAamp viral RNA minikit (Qiagen, Manchester, UK).

A sequence-independent single-primer (SISPA) approach was performed as described previously [18,19]. Reverse transcription was performed by mixing 4 μL of DNase-treated RNA and 1 μL of Primer A (5′-GTTTCCCACTGGAGGATA-N9-3′, 40 pmol/μL) [20], incubating for 5 min at 65 °C, then cooling to room temperature. First-strand synthesis was performed by adding 2 μL SuperScript IV First-strand Buffer, 1 μL 10 mM dNTPs, 0.5 μL 0.1 M DTT, 1 μL H_2_O and 0.5 μL SuperScript IV (Thermo Fisher, Loughborough, UK), followed by incubation for 10 min at 42 °C.

Second-strand synthesis was performed by adding 1 μL 5x Sequenase Buffer, 0.45 μL Sequenase dilution buffer, 3.85 μL H_2_O and 0.3 μL Sequenase (Affymetrix, High Wycombe, UK) prior to incubating for 8 min at 37 °C.

Amplification of cDNA was performed using 5 μL of the reaction as input to a 50 μL Q5 High-Fidelity reaction (New England biolabs) according to the manufacturer’s instructions, using 1 μL Primer B (5′-GTTTCCCACTGGAGGATA-3′, 100 pmol/μL) [20], with PCR cycling conditions of 98 °C for 30 s; 35 cycles of 98 °C for 10 s, 50 °C for 20 s and 72 °C for 2 min, followed by 72 °C for 2 min. Amplified cDNA was purified using a 2:1 ratio of AMPure XP beads (Beckman Coulter, Brea, CA, USA) and quantified by Qubit High Sensitivity dsDNA kit (Thermo Fisher), both according to the manufacturer’s instructions.

Illumina library preparation and sequencing was performed as previously [18]. Briefly, Nextera XT V2 kit sequencing libraries were prepared using 1.5 ng of amplified cDNA as per the manufacturer’s instructions. Samples were sequenced on a 2 × 150 bp-paired end Illumina MiSeq run.

Reads were trimmed to remove adaptors and low-quality bases using trimmomatic (0.3.0) with default parameters, to achieve an average phred score of Q30 across the read.

Reads were mapped to RVFV reference genome sequences (Genbank DQ380149.1, DQ380200.1, DQ375406.1) using BWA MEM (v0.7.17). Quasibam [21] was used to generate consensus sequences (using a 20x coverage cut off and mixtures greater than 20% coded as IUPAC ambiguities) and text files containing data on nucleotide frequency, depth and quality metrics. Alignment and analysis of nucleotide consensus sequences was performed using the ClustalW method in MegAlign (v11.0.13).

### 2.6. Challenge Study Design

In the first experiment, 50 female mice were used. Three groups of 15 animals were challenged with 100, 10 and 1 plaque forming unit (pfu) of mammalian cell-grown RVFV. An additional group of 5 animals were mock-challenged with phosphate-buffered saline (PBS; Gibco, Paisley, UK). At days 1 and 3 post-challenge, a pre-assigned group of 5 animals from each RVFV-challenged group were euthanised to assess responses at these timepoints. The remaining animals were used to assess survival, with the studies scheduled to last up to 21 days post-challenge.

A second study was performed with 50 mice using the same methodology as described above, with the exception of using insect cell-grown RVFV in replacement of mammalian cell group RVFV. In addition, 5 male and 5 female mice were also challenged with 10 pfu mammalian cell-grown RVFV to demonstrate reproducibility of the model and ascertain potential differences between sexes.

### 2.7. Challenge, Monitoring and Sampling

Prior to challenge, animals were sedated with the inhalational anaesthetic agent, isoflurane. The virus was subcutaneously inoculated into each of the rear hind limbs towards the tarsal joint (ankle) using a volume of 40 µL per limb.

Bodyweight and temperature were monitored daily, the latter via an indwelling temperature chip (identiCHIP). Clinical and behavioural scores were assessed at least twice a day, which was increased to four hourly periods between days 2–10 post-challenge, due to the rapid increase in severity of abnormal clinical and behavioural signs and onset of morbidity. Each sign was assigned a numerical value (1, eyes shut; 2, ruffled fur, aversion to light (photosensitivity); 3, abnormal posture (hunched or arched), lethargy; 5, laboured breathing; 8, incoordination; 10, immobility), which were summed to derive a total cumulative score at each monitoring timepoint.

At necropsy, samples of liver, spleen, kidney, brain, eye and ovary were placed into a PreCellys tube containing ceramic beads and stored at −80 °C for viral RNA measurement. In addition, these tissues were sampled consistently and placed into 10% neutral-buffered formalin (NBF) for pathological examination. Blood was collected via cardiac puncture, with 100 µL added to an animal RNAprotect blood tube (Qiagen, Manchester, UK) and stored at −80 °C for viral RNA measurement. The remainder was placed into a serum separation tube (SST; Becton Dickinson, Swindon, UK) with sera processed and stored at −80 °C for measurement of analytes by Luminex assay.

### 2.8. Viral RNA Measurement

Tissue samples for viral RNA analysis were weighed, resuspended in 1.5 mL PBS and homogenised using a PreCellys 24 homogeniser (Stretton Scientific, Alfreton, UK). A total of 200 µL of tissue homogenate or blood was transferred to 600 µL RLT buffer (Qiagen, Manchester, UK) plus beta-mercaptoethanol and mixed with an equal volume of 70% ethanol.

Tissues were further homogenised through a QIAshredder (Qiagen, Manchester, UK) at 16,000× *g* for 2 min and RNA was extracted by KingFisher Flex automatic extraction using the BioSprint 96 one-for-all veterinary kit (Indical, Leipzig, Germany) as per the manufacturer’s instructions. RNA was eluted in 100 µL AVE buffer (Qiagen, Manchester, UK). Samples were analysed by qRT-PCR using the TaqMan Fast Virus 1-Step Master Mix RT-PCR kit (Thermo Fisher, Loughborough, UK) with the fast-cycling mode and primers/probe targeting the G2 gene of RVFV M-segment (accession no. AF134508) [22]. Quantification of the viral load was determined using a 10-fold dilution series of RVF M-segment in vitro transcript from 1 × 10^7^ to 1 × 10^1^ copies per reaction.

### 2.9. Histopathological Analysis

Samples of liver, spleen, brain, kidney, ovary and eye fixed in 10% NBF were processed routinely into paraffin wax. Sections were cut to 4 µm and stained with haematoxylin and eosin (H&E). Slides were scanned digitally using a Hamamatsu S360 digital slide scanner and examined using ‘ndp.view2’ software (v2.8.24).

Additional sections were stained using the RNAscope technique to assess for the presence of RVFV RNA, as per previously published methodology [23], but using a 2.5LS Probe-V-RVFVZH501-NP probe (Catalogue no. 496 938, Advanced Cell Diagnostics).

A subjective scoring system was used to evaluate the presence and severity of microscopic pathological changes attributable to infection with RVFV in the H&E-stained tissue sections (minimal, mild, moderate and marked). Furthermore, the following scoring system was used to evaluate the degree of staining for viral RNA: 0 = no positive staining; 1 = minimal; 2 = mild; 3 = moderate and 4 = abundant staining.

All histological evaluations were undertaken by a qualified veterinary pathologist blinded to the animal and treatment details to minimise bias.

### 2.10. Luminex Analysis

A 19-plex mouse cytokine/chemokine panel was used consisting of granulocyte colony-stimulating factor (G-CSF), granulocyte-macrophage colony-stimulating factor (GM-CSF), interferon alpha-2 (IFNα2), interferon-gamma (IFNγ), interleukin(IL)-1β, IL-2, IL-4, IL-5, IL-6, IL-8, IL-10, IL-12(p70), IL-15, IL-17A, IFNγ-inducible protein 10 (IP-10), monocyte chemotactic protein 1 (MCP-1), macrophage inhibitory protein (MIP)-1a, MIP-1b and tumour necrosis factor alpha (TNF-α) (Millipore, Watford, UK). The assay was performed according to the manufacturer’s instructions.

After completion of staining, to remove plates from the CL3 laboratory for analysis, beads were treated with formalin, as previously reported [24,25]. Beads were resuspended with 100 μL/well of 10% formalin solution made by dilution of 100% formalin (40% *w*/*v* formaldehyde solution) (Scientific Laboratory Supplies, Nottingham, England) 1:9 *v*/*v* with phosphate-buffered saline solution (Thermo Fisher, Loughborough, England). Plates were fumigated with formaldehyde vapour overnight at room temperature for 16 h with the lids left ajar to allow vapour to reach all surfaces. Following fumigation, plates were removed from the CL3 laboratory and washed twice with wash buffer and once with sheath fluid in a Containment Level 2 laboratory to remove formalin solution before being resuspended in 150 µL of sheath fluid.

The results were acquired on a Luminex MAGPIX instrument using Exponent software (Invitrogen, Paisley, UK). At least 50 events per region were collected and median fluorescence intensity (MFI) measured. MFI values were converted to concentrations using results from a standard cytokine preparation.

### 2.11. Statistical Analysis

Statistical analyses were performed using MiniTab, v.16.2.2 (Minitab Inc., State College, PA, USA). A non-parametric Mann–Whitney statistical test was applied to ascertain significance between groups. A significance level below 0.05 was considered statistically significant.

## 3. Results

### 3.1. Sequence Comparison of RVFV Grown in Insect and Mammalian Cell Lines

Genome sequencing was undertaken using two technical replicates of the RVF virus preparations grown on insect and mammalian cell lines and compared to the reference genome sequence (Table 1). Differences between the reference and cultured genomes were observed at six sites: four in the M segment and one each in the S and L segments (Table 1). Although no majority base substitutions were detected between the reference genome and the virus preparations, nucleotide mixtures greater than 20% (coded as IUPAC ambiguities in Table 1) were detected at three positions in the mammalian-grown RVFV and three positions in the insect-grown RVFV.

### 3.2. RVF Virus Cultivated in Insect Cells Results in a More Rapid Disease Progression, but the Same Dose Is Required for All Animals to Meet Humane Clinical Endpoints, Compared to Mammalian Cell-Grown Virus

**Figure 1:**
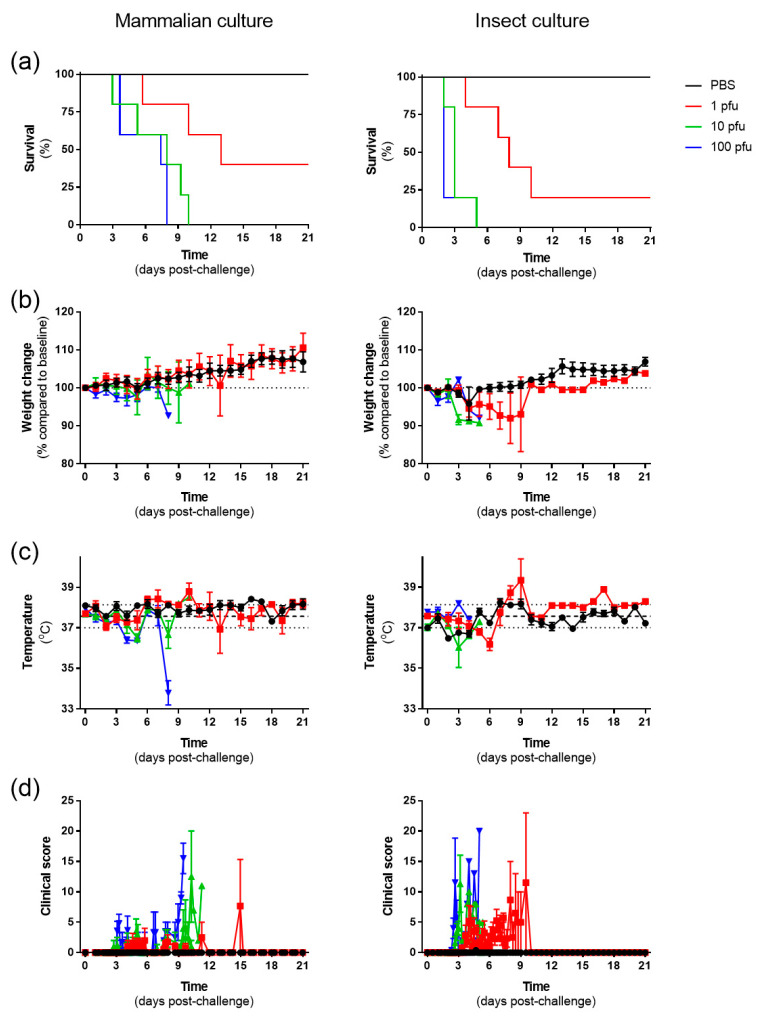
Survival and clinical observations. (**a**) Kaplan–Meier survival plots. (**b**) Bodyweight change as a percentage compared to day of challenge. (**c**) Body temperature recorded at the same time each day. Dashed line shows mean value of animals at baseline (*n* = 50) with dotted lines showing +/− standard deviation. (**d**) Clinical score represented as a cumulative total assigned for each sign recorded. (**b**,**d**): data points represent mean values with error bars denoting standard error. Black squares, PBS; red squares, 1 pfu; green up triangle, 10 pfu; blue down triangle, 100 pfu. *n* = 5 animals per group.

In an attempt to mimic natural infection from a mosquito bite, Balb/C mice were challenged via the subcutaneous route on each hindlimb adjacent to the tarsal joint using RVFV that had been passaged three times on mammalian cells (VeroE6) or insect cells (C6/36). Challenge doses of 10 and 100 pfu resulted in all animals meeting humane clinical endpoints, but these were delayed with the mammalian cell-grown virus compared with that cultivated on insect cells. A lower challenge of 1 pfu resulted in a subset of animals surviving with both virus preparations (Figure 1a). Disease progression consisted of bodyweight loss (Figure 1b), and prior to meeting humane clinical endpoint, a sharp drop in body temperature was observed in several animals (Figure 1c). A deterioration in clinical condition was often rapid; consequently, assessment frequencies were increased to every 4 hours during critical stages of the study to meet animal welfare considerations. For analysis, clinical scores were assigned a numerical value, and the results demonstrated onset of signs on day 2 post-challenge (Figure 1d). Scores were similar across the different virus preparations, with similar disease severity scores recorded immediately prior to animals meeting humane clinical endpoint criteria.

### 3.3. The Initial Virus Tropism Is to the Liver, before Becoming More Widely Disseminated throughout Other Tissues

**Figure 2:**
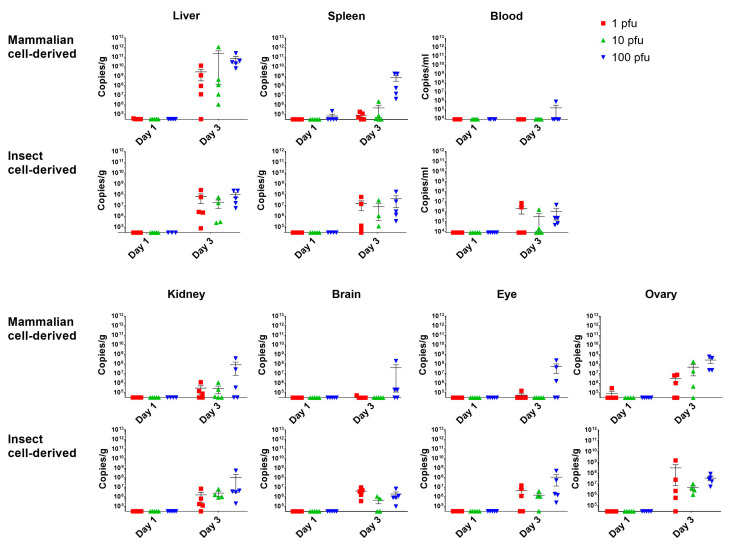
Viral RNA levels in the blood and tissues on day 1 and 3 post-challenge with RVF virus. Symbols show responses from individual animals with line and whisker plots denoting mean and standard errors. *n* = 5 animals per group.

To determine early events after RVF virus infection, five mice from each challenge group were euthanised on days 1 and 3 post-challenge, with blood and tissues processed to assess viral RNA levels. This revealed only sporadic, low-level detection in samples collected at day 1; however, by day 3 post-challenge, more broad detection was observed (Figure 2). Viral RNA levels were highest in the liver with both challenge virus preparations. For the insect cell-derived virus, detection was observed across most tissue, including the blood, indicating systemic spread. Due to the slower disease progression with the mammalian cell-grown virus stocks, differences were more noticeable. Secondary sites of viral RNA detection included the spleen, kidney and ovary, where it was detected following challenge with different doses and prior to detection in the circulation.

### 3.4. Histopathological Lesions Were Observed in the Liver and Spleen

**Figure 3:**
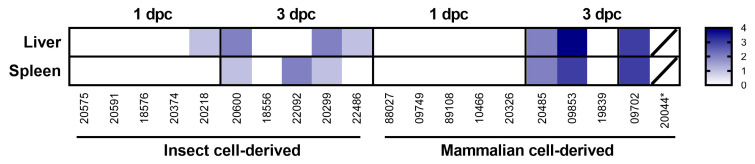
Heatmap representing the severity of microscopic lesions observed in the liver and spleen after challenge with 10 pfu RVF virus. Key: 0—normal, 1—minimal changes, 2—mild changes, 3—moderate changes, 4—marked changes. * found dead in the cage, samples not collected. Five-digit numbers refer to animal identification.

**Figure 4:**
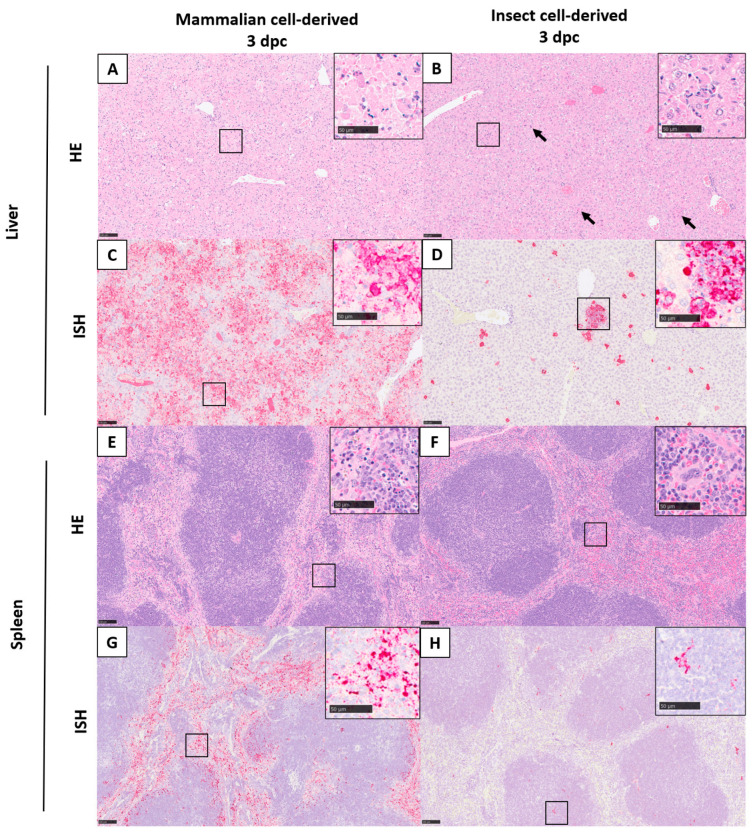
Microscopic lesions and viral RNA staining of animals infected with mammalian cell- (**left**) and insect cell-grown (**right**) RVF virus in the liver and spleen. Liver; multifocal hepatocyte degeneration and necrosis denoted by arrows (**A**,**B**) and viral RNA staining (**C**,**D**). Spleen; variable loss of mononuclear cells in the red and white pulp (**E**,**F**) and viral RNA staining (**G**,**H**). Inset, higher magnification images of the area highlighted by the smaller box in the main panel. HE, ISH. Scale bar denotes 100 μm for main panel and 50 μm for inset panels.

Microscopic lesions associated with infection with RVFV were noted in the liver and spleen of both the mammalian- and the insect-grown groups. These were largely absent at day 1 post-challenge but were observed at day 3. The severity of microscopic changes noted in the spleen and liver for individual animals are summarised in Figure 3 and representative images are shown in Figure 4.

In the liver, lesions comprised degenerating single or small foci of hepatocytes, characterised by shrunken cells with hyper-eosinophilic cytoplasm and nuclear pyknosis and karyorrhexis. At day 1 post-challenge, only one animal in the insect cell-derived group had minimal changes in the liver. By day 3 post-challenge, minimal to mild changes were noted in 3/5 animals in the insect cell-derived group and mild to marked changes in 3/4 animals in the mammalian cell-derived group.

In the spleen, lesions comprised degeneration and loss of mononuclear cells in both the red pulp and the white pulp and prominent tingible body macrophages (Figure 4E,F). Lesions were absent in all animals from both groups at day 1 post-challenge. By day 3, minimal to mild changes were noted in 3/5 animals in the insect cell-derived group and mild to moderate changes in 3/4 animals in the mammalian cell-derived group.

Lesions were absent in brain, kidney, ovary and eye of all animals from both groups throughout the time course.

### 3.5. Viral RNA Was Detected in a Range of Tissues

**Figure 5:**
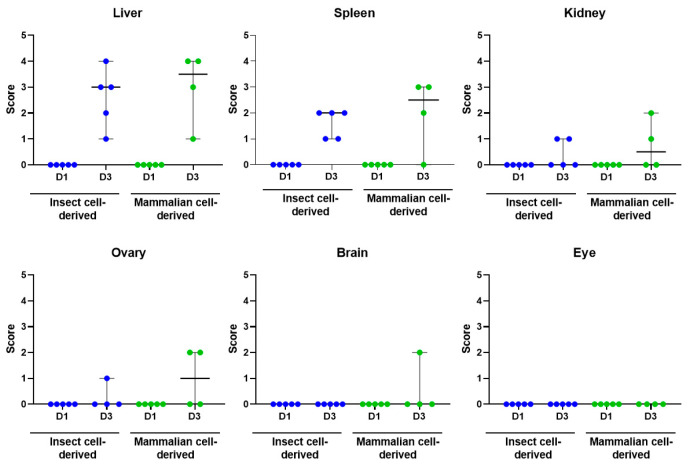
Scatter plots showing the degree of staining for RVFV RNA in the liver, spleen, kidney, ovary, eye and brain. Blue symbols denote insect cell-derived group and green symbols those challenge with mammalian cell-derived RVFV. Lines represent median plus 95% confidence intervals.

**Figure 6:**
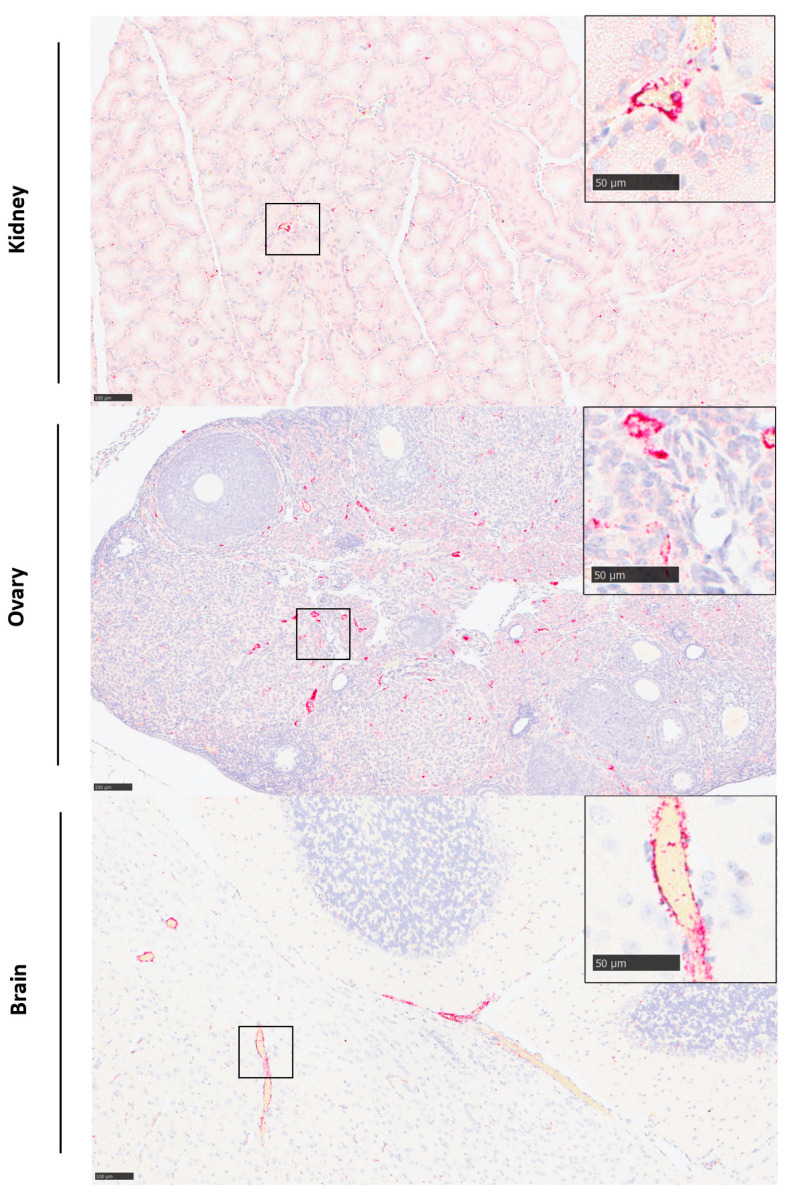
Staining for the presence of RVFV RNA in the kidney, ovary and brain of animal 09839 at 3 days after challenge with mammalian cell-derived virus. (**Top**): Kidney. Scattered staining of cells in the medullary interstitium. ISH. (**Middle**): Ovary. Scattered staining of cells in follicles and tunica albuginea. (**Lower**): Brain. Staining of vascular walls in larger vessels in the neuropil. Insets, higher power images of stained cells from the area highlighted by the smaller box in the main panel. ISH. Scale bar denotes 100 μm for main panel and 50 μm for inset panels.

Viral RNA was not detectable by in situ hybridisation at day 1 post-challenge but was detected variably in all tissues examined at day 3 (Figure 5). Staining was most prominent in the liver (Figure 4C,D) and spleen (Figure 4G,H). In the liver, staining was visualised primarily within hepatocytes, with severity scores ranging from one to four in all animals from both groups. Stained cells were observed in cells in both the white and red pulp of the spleen, with severity scores ranging from one to two in all animals in the insect cell-derived group and two to three for 3/4 animals in the mammalian cell-derived group. In the kidney, viral RNA was detected in two animals in the insect-grown virus challenge group (score 1) and two animals in the mammalian-grown group (score 1–2); staining was prominent in the glomeruli as well as cortical and medullary interstitium (Figure 6, top). In the ovary, viral RNA was detected in scattered cells in one animal in the insect-grown group (score 1) and two animals in the mammalian-grown group (score 2) (Figure 6, middle). In the brain, viral RNA was detected in one animal in the mammalian-grown group (score 2); staining was present primarily in blood vessel walls in the neuropil, meninges (Figure 6, bottom) and other vascular structures (choroid plexus) throughout the brain, as well as other scattered cells in the neuropil. Staining was absent in the eyes of all animals from both groups.

### 3.6. Cytokine, Chemokine and Growth Factor Levels Were Elevated after Challenge with RVF Virus

**Figure 7:**
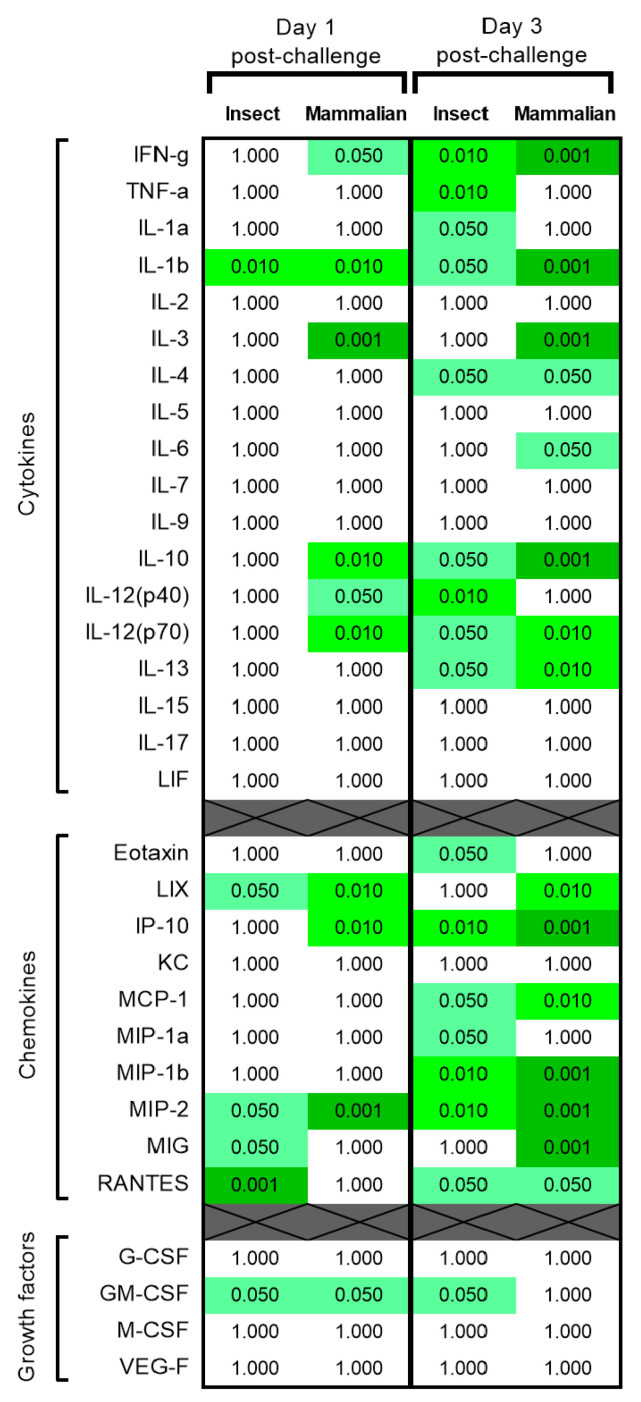
Statistically significant differences in cytokine, chemokine and growth factors concentrations in mice challenge with 10 pfu RVF virus compared to PBS control. Results show *p* values from Mann–Whitney statistical analysis. *n* = 5 animals per group. Where *p* > 0.05, a value of 1.000 has been assigned. Green coloured boxes denote statistical significance at a level less than the value contained within.

Sera prepared from blood samples collected 1 and 3 days post-challenge were assessed for biomarker levels. Differences were observed compared with the PBS control group for several analytes after challenge with 10 pfu (Figure 7). Cytokine levels of IFN-γ, IL-1β, IL-3, IL-10 and IL-12(p70) were elevated at day 1 post-challenge; these were maintained at significantly higher levels on day 3 post-challenge compared with controls. Insect cell-cultivated virus showed some differences compared with mammalian cell-cultivated virus, with levels of TNF-α, IL-1 and IL-12(p40) higher in the former compared with the latter. Similarly, chemokine levels differed between the two virus preparations. Only MIP-2 levels were consistently higher across both groups at day 1 and 3 post-challenge. Of the four growth factors measured, only GM-CSF was significantly elevated. The results from the 1 and 100 pfu challenge doses were also measured (Supplementary Figures S1–S3).

### 3.7. Challenge with 10 Pfu Mammalian Cell-Grown RVF Virus Is Reproducible and Results in a More Rapid Disease Progression in Male Mice

**Figure 8:**
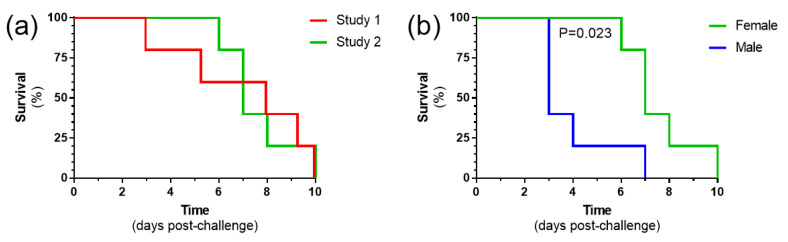
Survival analysis with 10 pfu mammalian cell-grown RVF virus. (**a**) Results from two independent studies with female animals. (**b**) Comparison of female and male animals within the same challenge study. *n* = 5 animals per group.

To assess reproducibility of the pathogenesis, a second study was conducted using the previously defined dose of 10 pfu, the lowest dose which uniformly resulted in all animals meeting humane clinical endpoints. Whilst our first challenge studies were conducted in female mice, an equal number of male and female mice (*n* = 5 per group) were used in this study. When female mice were compared between the two studies, all animals met humane endpoints by day 10 post-challenge, with no differences observed (*p* = 0.701, log-rank survival analysis) (Figure 8a). When the female and male mice were compared, the latter met humane endpoints more rapidly, which reached statistical significance (*p* = 0.023, log-rank survival analysis) (Figure 8b).

## 4. Discussion

The results described within demonstrate that passage methodology of viral stocks can exert an effect on the pathogenesis of subsequent virus infection in animal models. After three passages of RVFV in insect and mammalian cells, genetic differences from the reference genome were observed at three separate sites in both preparations. These changes were never full transitions and remained degenerate bases, indicating a significant level of variation within the viral population at these positions. The presence of mixed variants within the viral population could account for phenotype differences observed in the mice. This has been shown previously when a stock of ZH501 strain RVFV was demonstrated to contain an equal mix of two viral subpopulations; M847-G and M847-A. Upon inoculating mice with a virus containing either M847-G or M847-A, infection with the latter produced a more virulent phenotype. Furthermore, M847-A demonstrated increased fitness, evidenced by this variant quickly becoming the major viral population in mice that were inoculated with the M847-G variant [26]. In our study, we did not observe any changes at this position, with M847-A being present across both viral preparations. It is plausible that the mixed populations observed at different positions within the mammalian cell-grown and insect cell-grown RVFV genomes (Table 1) could influence infection kinetics within the mice. This could be investigated further in the future by comparing genomes from mouse tissues post-infection, to identify changes in subpopulations at the identified positions, followed up with subsequent investigation into their influence on phenotype.

With Dengue virus, proportions of the variant virus altered the following passage in a mosquito cell line compared with a mammalian cell line: different variants become dominant after serial passages [27]. Other groups investigating the effect of culture conditions on viral pathogenicity have also cultivated viruses for three passages under different conditions prior to assessing effects [28]. In addition, previous studies have demonstrated that after a single passage membrane glycosylation is acquired, it can be altered with no known impact on the viral genome [29].

With the insect cell-grown RVFV, animals met humane endpoints at earlier timepoints than those challenged with mammalian cell-grown virus. Nevertheless, the lowest dose resulting in all animals meeting humane endpoints after challenge via the subcutaneous route was the same at 10 pfu. This concurs with other groups where, following intraperitoneal challenge, RVFV established infections at doses ranging from 24 to 24,000 pfu [30]. After the subcutaneous challenge of mice, the LD_50_ for the same strain of RVFV used in this study, ZH501, has been reported to be 0.27 pfu [31] and 8.5 pfu [32]. Our results suggest a challenge dose of 10 pfu is sufficient to cause reproducible, uniformly severe disease. This is lower than that reported by others, where challenge doses of 1000 pfu are used [31,32,33].

The subcutaneous route was utilised to resemble infection via mosquito bite, the main route of transmission for ruminants [34]. For human exposure, infection from mosquitoes is rarer and is instead caused from direct contact with infected blood or tissues of infected animals [35]. Therefore, to specifically model the latter, an alternative challenge route may need to be considered.

During our studies, we observed a rapid deterioration in animals. This recapitulates findings from early studies with RVF virus where the interval between the onset of sickness and death never lasted more than 3 h [12]. The detection of the virus across different organs at 3 days post-challenge and highest levels observed in the liver align with those of animals challenged via the intraperitoneal [30,36] and subcutaneous route [31,33]. Others have also shown in mice challenge studies that RVFV was not detected in blood or tissues until day 2 post-infection [32]. The discovery of the virus in the brain is in line with other studies [30,31,32]. The route of this neuro-invasion was not established, but evidence indicated it was either via olfactory nerves leading to infection of the olfactory bulbs or ascending infection of cranial nerves into the brainstem [32].

From analysis of cytokine, chemokine and growth factors levels, at day 1 post-challenge, IL-1β, LIX, MIP-2 and GM-CSF levels were raised in both the insect cell- and mammalian cell-grown virus groups. A broad immunological profile was demonstrated at day 3 post-challenge with cytokines from both Th1 and Th2 subsets being elevated, supporting a previously described hypothesis of a strong unregulated immune response [33]. Our results align with a RVFV encephalitis model using strain CC057 mice, where levels of IL-10, IP-10, IL-6, MIG and MCP-1 were shown to be elevated in plasma on days 5–8 post-infection [37]. Interestingly, the increased concentration of the chemokine MCP-1 has previously been associated with vascular leakage in Dengue virus infection [38]. Future work assessing whether this is the same mechanism for RVFV is warranted.

The observation of different disease kinetics from Infection with the two virus preparations in the mice concur with reports from the experimental infection of goats [39]. A number of factors may account for this host cell effect. The first may be the glycosylation profiles of progeny viral particles produced in the two cells lines: insect and mammalian. Whilst host factors for RVFV are poorly defined, recent data provide evidence for lipoprotein receptor-related protein 1 (Lrp1) as a host entry factor where the Gn interacts, allowing proteinaceous entry of the virus into the cell [40]. Ross River virus has been shown to exert different responses dependent on the cell line used for cultivation due to differences in viral envelope N-linked glycosylation with that grown on mammalian cells, eliciting robust early antiviral responses in the skin compared to that grown in mosquito cells after the intradermal challenge of mice [14]. The mosquito cell-grown virus had a longer disease progression, with higher clinical disease scores and a longer time for disease resolution compared to those challenged with mammalian cell-grown virus [14]. Likewise, virus-like particles (VLPs) derived from Mayaro virus have been produced in mosquito and mammalian cells and tested for immunogenicity in mice, with the latter inducing higher neutralising antibody titres [41]. A similar difference with Chikungunya VLPs propagated in human and insect cells has been demonstrated in respect of N-linked glycosylation profiles [15], but after immunisation of guinea pigs, similar neutralising antibody responses were observed [42]. Therefore, different glycans may impact correct antigen folding and stability in a pathogen-specific manner. Further evidence supporting this is that RVFV derived from insect cells has been suggested to interact with C-type lectin receptors (CLR) differently to those grown in mammalian cells [43].

Another plausible explanation is that during culture of RVFV in different cell lines, other proteins may be expressed at different levels. For example, the expression of a non-structural protein on the S segment (NSs) has been shown to be affected by culture conditions. Some reports demonstrate NSs expression in the mosquito C6/36 cell line [44], whereas others show low or no expression in insect cells as compared to mammalian cell lines [45,46]. The NSs functions as an interferon antagonist [47] and induces degradation of the RNA-dependent polymerase [48], and thus is a major virulence factor. The RVFV 78 kDa protein (P78), a membrane glycoprotein, has been shown to be incorporated into virus grown in C6/36 cells but not in mammalian (Vero E6) cells [49] and has been shown to provide attenuation in mice [50].

Finally, the temperature of cultivation may also exert an effect. It has been reported that Dengue virus cultivated on C6/36 cells was more virulent in a mouse model when grown at a higher temperature (35 °C) compared with culture at 28 °C [28]. Therefore, as the Vero E6 cells were grown at 37 °C and the C6/36 at 28 °C in this study, there is a similar temperature difference between the two stocks.

During these studies, we were interested in determining whether the sex of the animals played a role in their susceptibility to infection, especially as experiments studying the pathogenesis and kinetics of RVFV mainly only use female mice [30,32]. Female mice have been demonstrated to have higher serum immunoglobulin levels compared with males, including IgG2b levels both within the total serum component but also among virus-specific antibodies [51,52]. Our results show that male mice succumbed to infection quicker than female counterparts. This contrasts with other reports, where using a panel of wild-type mouse strains, sex differences in RVFV survival were not observed after challenge with a virus produced via a reverse-genetics system, with the exception of A/J mice where, in contrast with our results, males succumbed approximately 1 day later than females [53]. Similarly, no differences were observed in RVFV survival curves between male and female animals when four collaborative cross-strains were evaluated [37]. However, with these studies, the challenge virus was generated using a reverse-genetics system in contrast with growth by passage on cell lines.

Sex-specific susceptibility to other viral pathogens has been reported. For example, studies in mice infected with Zika virus at an early development stage reported that male mice exhibited greater behavioural deficits and neuropathological abnormalities than female littermates [54]. This may be due to differences in microglial number and chemokine expression profiles observed during critical development periods in rodents between the two sexes [55]. After infection with coxsackievirus B3, CD4+ T cells from male animals predominantly produced the Th1 cytokine IFN-gamma, whereas those from females produced the Th2 cytokine IL-4 [56]. With the same infection, a dramatic sex difference in the effects of TLR signalling in the T regulatory cell response has been reported [57]. Whilst male animals are more susceptible to the aforementioned infections, with other pathogens, greater mortality is seen with female mice, such as when three DNA viruses were assessed: herpes simplex virus type I, murine cytomegalovirus and vaccinia virus [58].

The reasoning for differential susceptibility between sexes might be multifactorial. One aspect may be differences in the immune system. CD8+ T cells from female mice have been shown to preferentially become short-lived effectors in comparison with those from male mice, which have a propensity to give rise to more memory precursor effector cells. This is likely due to female CD8+ T cells exhibiting an enhanced capacity to respond to IL-12 [59]. After influenza A challenge, a sex bias in invariant natural killer T cells has been observed, with female mice expressing higher numbers in the lung and liver compared to males [60]. Similarly, protection from influenza A virus in female mice has been reported through suppression of inflammatory responses [61] and significant differences in the systemic and pulmonary “redox profiles”, including female animals having a higher total antioxidant power in serum and lungs [62]. A separate study contradicts these earlier studies and instead suggests that female mice undergo more severe disease than male mice after infection with different influenza A subtypes [63].

Another explanation is derived from sex-based differences in SARS-CoV mouse studies, with male mice being more susceptible, having been shown to be independent of T and B cell responses, but instead indicating a role for oestrogen receptor signalling [64]. A similar effect on receptor expression has been shown with SARS-CoV-2, as in both mice and humans, aged males have been shown to have elevated expression of ACE2 across organs [65]; this is likely due to its sexually dimorphic expression dependent on sex chromosomes and hormones [66]. Similarly, many immune genes and factors involved in the responsiveness of immune cells to female (oestrogen/progesterone) and male (testosterone) hormones have X-linked expression [67]. This has been observed in challenge studies, with aged male mice (2 years old) showing more severe disease manifestation after SARS-CoV-2 challenge than female counterparts [68]. The female hormone, oestrogen, when tested in vitro, has been shown to reduce ACE2 expression on differentiated airway epithelial cells [69].

Our results show the importance for testing medical countermeasures against RVFV in both male and female animals, in line with others [70]. Indeed, for the influenza A vaccine, there are differences in immune responses between the sexes, with female mice generating higher antibody responses and having increased protection against viral challenge than male animals [71].

In summary, our results provide evidence that the disease progression after RVFV challenge is affected by the cell source and culturing conditions of the viral inoculum and the sex of the animals. These results provide further avenues for exploration to establish the potential mechanism(s) involved with these differences and highlight the importance in establishing disease parameters when developing in vivo models of disease in order for data generated in subsequent studies to be fully interpreted.

## Figures and Tables

**Table 1:** Sequence changes in RVF virus cultivated on mammalian and insect cell lines.

Genome Segment	Segment Position	Mammalian Cell Base	Insect Cell Base	Reference Genome Base ^1^
S	522	A (99.9%)	W (78.8% A/21.2% T)	A
M	273	G (1.3% A/98.7% G)	R (43.95% A/56.05% G)	G
M	843	Y (45.8% C/54.2% T)	T (99.95%)	T
M	855	G (2.9% A/97.1% G)	R (46.55% A/53.45% G)	G
M	1315	Y (76.6% C/23.4% T)	C (99.95%)	C
L	5739	Y (24.05% C/75.95% T)	T (100%)	T

^1^ Reference sequence: S segment, DQ380149.1; M segment, DQ380200.1; and L segment, DQ375406.1.

## Data Availability

The data presented in this study are available on request from the corresponding author.

## Supplementary Materials

The following supporting information can be downloaded at: https://www.mdpi.com/article/10.3390/v15122369/s1, Figure S1: Levels of cytokines; Figure S2: Levels of chemokines; Figure S3: Levels of growth factors.
