# Supplementary material for: Pathogenesis of Rift Valley Fever Virus in a BALB/c Mouse Model Is Affected by Virus Culture Conditions and Sex of the Animals
Source: Viruses. 2023 Nov 30;15(12):2369. doi: 10.3390/v15122369 (PMC10747589; doi:10.3390/v15122369)
Supplement: Supplementary file 1 [file viruses-15-02369-s001.zip › viruses-2725283-supplementary.pdf]

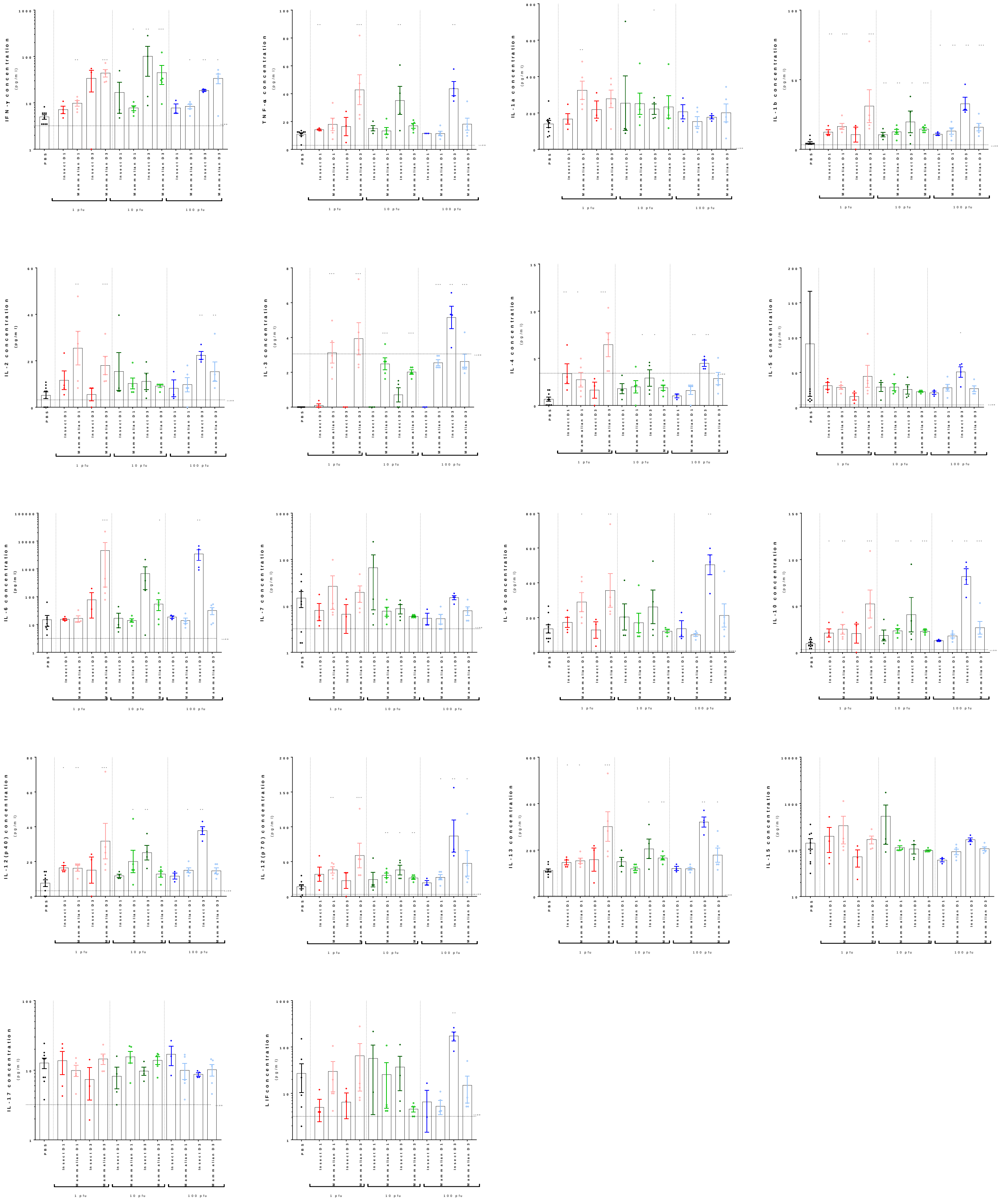

Supplementary figure S1. Cytokine levels in animals challenged with 1, 10 or 100 pfu insect or mammalian cell derived RVFV on day 1 and 3 post-challenge. Significant difference to PBS control group denoted by * (P<0.05), ** (P<0.01) or ***(P<0.001).

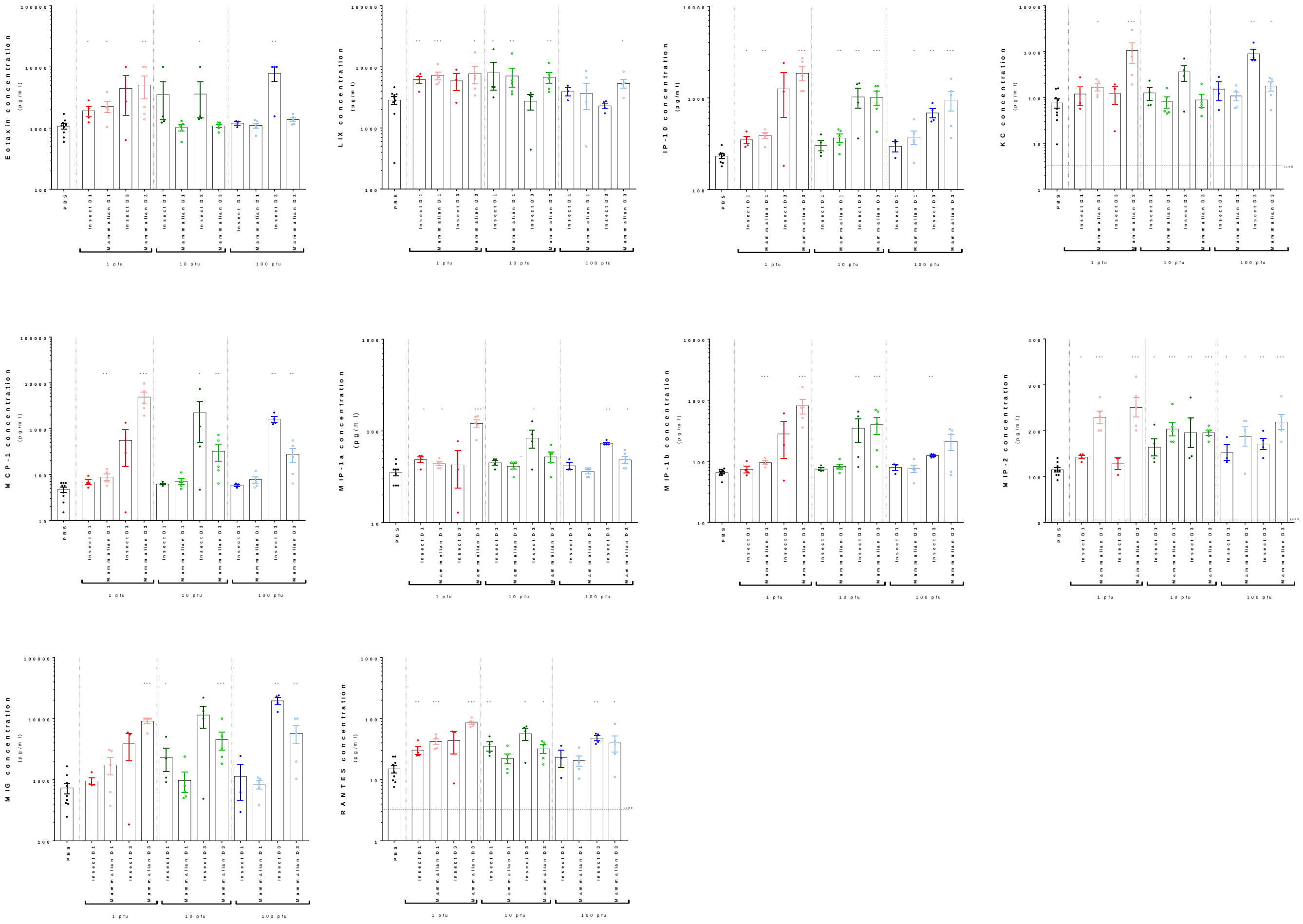

Supplementary figure S2. Chemokine levels in animals challenged with 1, 10 or 100 pfu insect or mammalian cell derived RVFV on day 1 and 3 post-challenge. Significant difference to PBS control group denoted by * (P<0.05), ** (P<0.01) or ***(P<0.001).

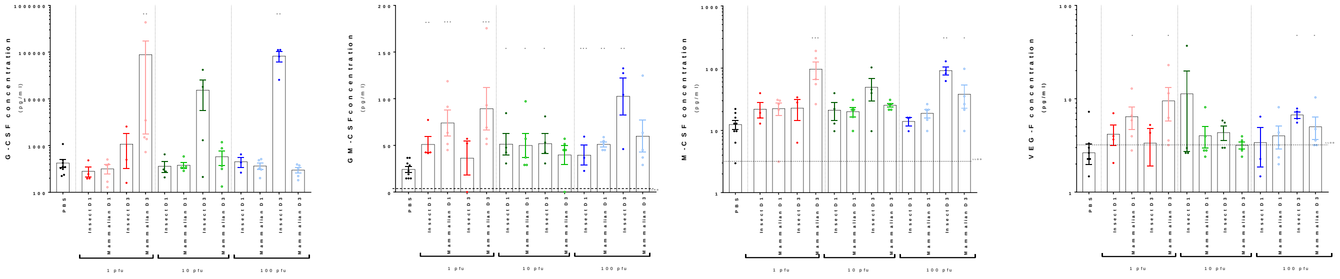

Supplementary figure S3. Growth factor levels in animals challenged with 1, 10 or 100 pfu insect or mammalian cell derived RVFV on day 1 and 3 post-challenge. Significant difference to PBS control group denoted by * (P<0.05), ** (P<0.01) or ***(P<0.001).
